# Rural healthcare professionals’ participation in Medical Assistance in Dying (MAiD): beyond a binary decision

**DOI:** 10.1186/s12904-024-01440-4

**Published:** 2024-04-25

**Authors:** Monique Sedgwick, Julia Brassolotto, Alessandro Manduca-Barone

**Affiliations:** Faculty of Health Sciences, University of Lethbridge, Lethbridge, AB T1K 3M4 Canada

**Keywords:** Assisted death, Medical assistance in dying, Palliative care, End-of-life, Healthcare professionals, Nurses, Physicians, Nurse practitioner, Clinical ethicist, Legislation

## Abstract

**Background:**

Medical Assistance in Dying (MAiD) was legalized in Canada in 2016 and amended in 2021. At the time that this study was conducted, the federal government was considering expanding the eligibility criteria to include patients whose death was not reasonably foreseeable. The purpose of this study was to better understand rural healthcare professionals’ experiences with assisted dying set against the backdrop of legislative expansion.

**Methods:**

A qualitative exploratory study was undertaken with general rural practice physicians, nurse practitioners, registered nurses, ethicists, patients, and patient families in rural Southern Alberta, Canada. For this paper, data from 18 audio-recorded and transcribed semi-structured interviews with healthcare professionals were analyzed using thematic analysis. Categories and patterns of shared meaning that linked to an overarching theme were identified.

**Results:**

Between the binary positions of full support for and conscientious objection to assisted dying, rural healthcare professionals’ decisions to participate in MAiD was based on their moral convictions, various contextual factors, and their participation thresholds. Factors including patient suffering; personal and professional values and beliefs; relationships with colleagues, patients and family, and community; and changing MAiD policy and legislation created nuances that informed their decision-making.

**Conclusions:**

The interplay of multiple factors and their degree of influence on healthcare professionals’ decision-making create multiple decision points between full support for and participation in MAiD processes and complete opposition and/or abstention. Moreover, our findings suggest evolving policy and legislation have the potential to increase rural healthcare professionals’ uncertainty and level of discomfort in providing services. We propose that the binary language typically used in the MAiD discourse be reframed to reflect that decision-making processes and actions are often fluid and situational.

**Supplementary Information:**

The online version contains supplementary material available at 10.1186/s12904-024-01440-4.

With the legalization of Medical Assistance in Dying (MAiD) in 2016, Canadians under specific and clearly defined legal and regulatory circumstances have a new option in end-of-life care beyond that offered through palliative care. Consequently, registered nurses, physicians, nurse practitioners across Canada have been directly impacted by changes made to the Criminal Code for MAiD. Given the significant social and medical changes this legislation presents, and considering their duty of care, it is perhaps not surprising that the element of choice can be overwhelming for health care professionals (HCP) who may find themselves at a crossroads of having to determine whether they will participate in the provision of MAiD.

Choosing to participate in MAiD becomes even more complex in rural settings where the intricacy of the rural context complicates HCPs’ decision-making to act in accordance with their personal and professional ethics. These intricacies include dual roles where personal and professional roles and responsibilities intersect, complex power dynamics between healthcare team members and organizational leaders, limited resources, close-knit communities, geographic isolation, challenges pertaining to privacy and confidentiality, and possible personal and professional repercussions for being involved with MAiD [1, 2].

To add to the complexity of deciding to what degree rural HCPs will be involved with MAiD, the current lay discourse suggests that decision-making regarding the provision of MAiD services for HCPs is a binary decision: one is either for or against MAiD and engages in corresponding actions (i.e., participation or nonparticipation). The decision to participate or not in MAiD can be relatively straightforward for some rural HCPs, but for others, it is a complex, uncertain, and evolving decision-making process. For some HCPs, willingness to participate in MAiD may be contingent on certain contextual factors that influence service delivery or on their own personal comfort with providing MAiD to certain populations or in certain circumstances. Consequently, because of its sensitive nature and continued legislative evolution, understanding HCPs’ experiences of participating or opting out of MAiD is crucial to ensuring high-quality accessible end-of-life care in rural settings.

The purpose of this paper is to present the experiences of rural HCPs (general rural practice physicians, nurse practitioners, registered nurses, and ethicists) with MAiD in rural Southern Alberta, Canada. Similar to Brown et al’s work [3], we found that there were systemic, relational, and contextual factors and a dynamic participation threshold that influenced HCPs’ decisions to participate in MAiD. For our participants, these factors included: (1) patient suffering; (2) personal and professional values and beliefs; (3) relationships with colleagues, patients and family, and community; and (4) changes in MAiD policy and legislation. Based on our analysis and understanding of the data, we suggest that participants engage in dynamic decision-making that results in actions along a continuum from full participation in the provision of MAiD services to nonparticipation. Last, we conclude by advancing an argument in favor of changing the existing binary language often found in policies, reports, guidelines, and the popular media, so that discourses might become more inclusive and open to the various positions HCPs may hold about MAiD at any given time.

## Background

The following section describes the context of the health zone in Alberta Canada where MAiD provisions are allowed and in which this study was conducted. We provide a description of rural practice since our focus is on rural HCPs’ experiences within the MAiD context.

## South zone & MAiD in Southern Alberta, Canada

Established in 2008, Alberta Health Services (AHS) is comprised of five health zones (South Zone, Calgary Zone, Central Zone, Edmonton Zone, and North Zone). It provides health care services to over 4.4 million Albertans as well as some citizens in British Columbia, Northwest Territories, and Saskatchewan. The South Zone is home to approximately 282,000 Albertans who are served by two regional hospitals and smaller rural hospitals [4]. Although part of a provincial healthcare system, AHS leaders suggest there are intraprovincial differences between urban and rural communities related to knowledge and attitudes about MAiD [5].

Regulatory bodies for physicians, nurses, and pharmacists in the province are responsible for developing and enforcing professional standards, protocols, and guidelines which provide direction for their members [6]. In Alberta, physicians, and nurse practitioners (NP) may assess and**/or** provide MAiD to patients who meet legislative criteria. If a physician or NP receives a request and no longer wishes to participate in the process, then within seven days, they must report the request to the MAiD Care Coordination Service to make a formal referral to transfer the patient’s care to another healthcare provider [7]. According to the College of Physicians and Surgeons of Alberta, eligibility for MAiD is determined through at least two assessments:1) an initial assessment that is conducted by the physician managing the patient’s care or arranged by the AHS Care Coordination Service and 2) an independent assessment that is arranged by the physician managing the patient’s care or the AHS Care Coordination Service [7]. There is no requirement that one of the assessors be the provider. Participation in assessment for MAiD does not necessitate participation in the provision, nor does the provider of MAiD need to have been involved in the assessment. However, any physician or NP who has had a patient transferred to them for the purposes of MAiD provision must verify that the patient meets the mandatory requirements and has provided informed consent.

Registered nurses (RNs) without the NP professional designation have a more limited role in the provision of MAiD services whereby they provide pre/post provision care. From 2016 to 2023, a total of 3,914 MAiD activities (activities are undefined but may include inquiries, assessments without provisions, and assessments with subsequent provision) have occurred with a total of 977 provisions have been administered throughout Alberta. Of the 977 provisions, 328 have occurred in the South Zone [8].

The AHS MAiD Care Coordination Service is a single point of contact for patients, families, and healthcare providers across the province. Each healthcare zone has a coordinator (typically a registered nurse) who is the initial contact person for patients, family members, staff, assessors, and providers. The coordinator conducts consultations for MAiD and organizes the assessments and provisions. Coordinators also complete required administrative documents, maintains lists of drugs recommended for use in the provision of MAiD, and provides education about MAiD and end-of-life services and supports [5].

## Changes in legislation

In September 2019, the Government of Canada introduced Bill C-7 in response to the ruling in the Truchon v Canada case in which the “reasonably foreseeable death” criterion (an eligibility requirement under in Bill C-14) was deemed unconstitutional. As a result, the Canadian Criminal Code was amended to permit MAiD for individuals who are experiencing grievous and irremediable suffering due to illness or disability, but whose natural deaths are not reasonably foreseeable. The amendment also includes a 90-day reflection period for patients whose death is not reasonably foreseeable and a waiver of final consent in particular circumstances [7]. This created a distinction between Track 1 cases (in which death is reasonably foreseeable) and Track 2 cases (in which it is not). In early 2024, the Federal Government extended the temporary exclusion of eligibility for MAiD applicants whose sole underlying medical condition is a mental illness until March 2027 [9].

### Characteristics of rural practice

According to some authors, the role of rural and remote RNs has not changed significantly in the last couple of decades and so they continue to be described as ‘expert generalists’ [10–12]. Since nursing practice becomes more generalized the smaller the rural hospital, there is an impermanence to their scope of practice which seems to expand to meet the needs of the community and in response to changes in legislation, regulation, and education requirements [10, 13–21]. Like rural RNs, general rural practice physicians also have a broad scope of practice, which increases as the degree of rurality increases [22]. Although general rural practice physicians report performing a wider spectrum of procedures (including providing obstetrical and emergency care and spending more time on patient care and on-call than their urban counterparts) medical programs in Alberta that incorporate rural practice placements within their curricula offer limited exposure to palliative care [23]. Though patients who qualify for MAiD have the right to access MAiD, for medical students who have limited exposure to end-of-life options and choices in their educational programs, they may encounter more uncertainty and ethical conflict when confronted with a request for MAiD, even though *all* HCPs in Alberta Canada can refer patients to the AHS MAiD Care Coordination Service.

### Literature review

In 2014, Lachman [24] suggested that without the option of refusal to fulfill a legal duty due to ethical values, religion, or ideological perspectives, that is, conscientious objection, the moral integrity of nurses within the realm of physician-assisted suicide would be compromised. Three years later, and one year after MAiD was legalized in Canada, Collins and Leier [25] suggested MAiD legislation could potentially erode the patient/physician relationship, disrupt continuity of care, and lead to subpar provision of care in rural and remote areas. According to these authors, such negative consequences were possible because of the limited number of physicians and allied healthcare professionals practicing within these settings and because they had little training in the provision and management of MAiD. Continuing in this vein, Schiller [26] outlined key components of MAiD legislation that could present challenges for nurse practitioners in rural and remote communities where accessibility to basic health care remains a struggle even to this day.

While this early research has helped in setting the foundation for what is known about MAiD in rural settings, the work of exploring and understanding the experience of MAiD participation and its moral implications among rural HCPs is in its infancy and is predominantly found in the nursing literature. To that end, the research studies we were able to locate were all qualitative and included participants who worked across a variety of practice settings including critical care, palliative, hospice, community, long-term care, urban and rural practice settings [3, 27–32]. In their study, Lamb et al. [27–29] explored the meaning of conscience for nurses within the context of conscientious objection. They found that when nurses encounter issues of conscience, staying true to their conscience was challenging especially if they work in a jurisdiction like Alberta, where mandatory referral was expected of them. These researchers questioned to what extent HCPs’ conscience could be professionally challenged before it became a violation of their freedom of conscience, which is protected under the law. Pesut et al. [30, 31] interviewed 59 registered nurses across Canada regarding their moral experiences with MAiD. These nurses used moral waypoints to make sense of their decision to participate in MAiD. Moreover, their decision to participate in MAiD was influenced by their relationships with family, friends, and colleagues. For those participants who practiced in rural settings, not compromising the trust and their relationships within the community influenced their decision to participate or not in MAiD. The researchers concluded that the diverse clinical conditions of patients who were eligible for MAiD may mean nurses are on unfamiliar ground, making it difficult for them to identify as conscientious objectors [33] potentially placing them between a rock and a hard place [30]. These conclusions are important given the recent and ongoing evolution of MAiD legislation.

In their qualitative study with physicians and NPs across the province of Saskatchewan, Canada, who choose not to participate in MAiD, Brown et al. [3, 32] found that endogenous factors (factors that originated within the participants) and exogenous factors (factors that originated external to the participants) influenced their decision to be nonparticipants. These authors concluded that participants needed to reconcile that is, harmonize the endogenous factors and different MAiD participation thresholds (care participation is possible but not MAiD provision and care participation is not possible beyond facilitation in referral). The authors advance the idea that within the conscientious objection discourse, two overlapping concepts exist: conscientious objections *to* MAiD and nonparticipation *in* MAiD.

Beuthin et al. [34] completed a qualitative narrative inquiry with 17 nurses (registered nurses, nurse practitioners, and licenced practical nurses) in British Columbia, Canada, who directly provided assistance in a MAiD provision, were involved in some aspect along the patient’s journey (e.g., provided information, acted as a witness to the medical assessment, provided care before and/or after), or who had no patient involvement and identified themselves as conscientious objectors. Although most of these participants viewed MAiD as part of their professional responsibility for the provision of holistic non-judgemental care, some nurses described their experience as an ‘in-between’ space. Not linked to religious, spiritual, or ideological reasons, their uncertainty was rooted in fear and confusion around the legal, ethical, and professional implications, and lack of confidence. Based on their findings, Beuthin et al. concluded that, for these participants, sense-making about MAiD occurred on a continuum.

Consistent with these Canadian studies, researchers from other western countries have found similar findings regarding the complexity of conscientious objection. In an Australian study of physicians, Haining and Keogh [35] postulate that based on the strength of the individual’s conscientious objection, physicians fit on a continuum of complicity that is contingent upon their interpretation of the moral acceptability in participating in the voluntary assisted dying process. Haining and Keogh suggest that institutional guidance and education that explicitly explains how physicians can effectively preserve and protect their moral integrity while ensuring patients’ access is not impeded should be offered.

In a qualitative study with nurses, pharmacists, and social workers, Mills et al. [36] found that all the participants in their study viewed MAiD as a form of care nestled within a complex choreography of social discourses and moral logics well beyond a simple dichotomy of “choice versus care” (p.61). From this review, it appears that HCPs moral experiences within the MAiD context are varied and complex and that their decisions whether or not to support/participate in MAiD is not binary in nature. We aim to add to this existing body of literature with a focus on rural Canadian HCPs’ experiences of MAiD.

### Methods

Our qualitative exploratory study was undertaken with general rural practice physicians, NPs, RNs, ethicists, patients and patient families in southern Alberta, Canada. In this paper, we present HCPs’ participation in MAiD. Since little empirical research was previously undertaken exploring MAiD in rural Alberta, this design was appropriate [37] and allowed our team to develop a deeper understanding of related issues and experiences of the rural interdisciplinary team. Ethics approval was received from the University of Alberta, Edmonton, Canada.

All participants provided written or verbal informed consent. Participant names and potentially identifying information have been removed to the greatest extent possible to protect anonymity. Participant code numbers were assigned to each participant interview. Given the relatively small number of HCPs participating in the MAiD program in rural southern Alberta, we informed participants about the potential limits to confidentiality. To be more concise, the general rural practice physicians who participated in this study are referred to as ‘Physician’ and because of the small number of rural NPs and to enhance anonymity, we refer to NP participants as ‘Nurse’. None of the participants withdrew from the study.

### Data collection and analysis

We conducted qualitative semi-structured interviews between September 2021 and April 2022. Interviews with 9 nurses, 7 general rural practice physicians, and 2 clinical ethicists, lasting between 35 and 75 min were completed. The interviews were virtual via Zoom or via telephone, audio recorded, and transcribed verbatim. The interview guide developed for this study (see Supplementary information for the complete guide) included questions addressing participants’ experiences, challenges and rewards with being involved with MAiD, and their knowledge of legislation and policies. Consistent with qualitative interviewing, as the interviews progressed some questions were removed while others added. For example, since some participants spoke of ‘dual roles’ we asked the following question: “There are scholars who’ve expressed concerns that MAiD service provision might play out differently in rural areas because of things like healthcare professional shortages, dual roles where your personal and professional life might intersect, limited privacy or anonymity, and geographic isolation. Based on your experience, how warranted are these concerns?”

We used purposive sampling to recruit participants from several categories: general rural practice physicians and NPs who provide assessments and/or provisions, clinical ethicists who conduct MAiD-related consults, and RNs without the NP designation who provide care for MAiD patients and family members. Other inclusion criteria developed for this study included: being over 18 years of age, English speaking, and having worked in their position for a minimum of six months. Key contacts in AHS shared information about our study with individuals who met our inclusion criteria.

We analyzed our data using the thematic analysis process described by Braun and Clarke [38–40] which includes developing categories and interpreting patterns of shared meaning linking central ideas to an overarching theme. Each research team member read and re-read the transcripts, highlighted initial codes by identifying interesting features of the data, and looked for patterns. Potential categories were generated. We then met as a team to review, refine, and name the categories. Trustworthiness was supported by concurrent member checks during interviews, researcher reflection, and by achieving consensus among the research team [41]. The findings presented in this manuscript describe factors that influence the participants’ decision-making regarding MAiD, which created a continuum between supporting and participating in MAiD on one end and nonsupport and nonparticipation on the other. All of our participants were MAiD supporters to some extent, that is, they were not conscientious objectors and were supportive of the legalization of assisted dying, but their willingness to participate in assessments and provisions shifted along this continuum depending on contextual factors.

### Findings

When discussing their experiences, many participants spoke about the factors that inform their decisions to participate in MAiD, as well as what their participation/nonparticipation might look like for them under different circumstances. Like Brown et al’s [3, 32] findings, the influence each of these factors might have had on the participants’ decision-making processes and participation varied depending on the specific situation. Consequently, their decision-making processes were dynamic, situational, and reflective of a continuum.

#### The continuum

A continuum is a range or series of things that are slightly different from each other and that exist between two different possibilities. For this study, we envision the MAiD continuum as extending from one end with full support and full participation in MAiD (that is, engaging in assessments and provisions, in Track 1 and Track 2 cases) to another end with full oppposition and nonparticipation (that is, no engagement in the MAiD process resulting in referring the patient to another provider). Between these end points, there are multiple points made up of varying degrees of support and corresponding actions.

Our data confirm that the participants’ decisionsabout participation in MAiD were indeed scattered along such a continuum. For example, there were instances when providing information was considered acceptable but doing the provision was not. At other times, doing the provision was deemed more acceptable than the assessment because of perceived level of expertise.

*I think that for most physicians, it’s very uncomfortable – essentially, you’re killing somebody; you’re murdering somebody and to have that burden is, for most people, not something that they want to engage. So, I have lots of physician friends who are willing to do the assessments and feel very comfortable with that, but I think there’s only three or four of us that will do provisions (Physician 2).*

*I haven’t had a lot of palliative care training, you know, predicting somebody’s time before they die. It was a little uncomfortable for me to go and do assessments, and so I primarily got involved with the idea of delivery (Physician 6).*

The HCPs in our study also understood that conducting patient assessments and/or provision of MAiD was voluntary and was based on their objection to and moral acceptability of the various stages of assisted dying processes. As a result, the participants experienced shifts in their decision-making processes regarding support for and participation in MAiD suggesting that a threshold of participation acceptability existed for them. Thus, having a choice meant decisions and actions were dynamic.

*We talked a lot about the way it’s [referring to the MAiD program] been rolled out and that this is both a personal and professional issue. You are allowed to say ‘yes’ to participate, and you don’t have to justify that to anyone. And you can say ‘no, I don’t want to be involved,’ and you don’t have to justify that to anyone. And you can say ‘I’m not sure: maybe I’ll say ‘yes’ this time, and maybe, I’ll say ‘no’ next time.’ Just because you said no this time doesn’t mean you’ll always say no. You might say yes and just because you said yes this time, doesn’t mean you’ll say yes all the time. It’s not a final yes or no. It’s where you’re at. So, there have been providers that did opt out of a provision at a certain time, just because for whatever reason they weren’t in a space to say yes, or maybe they were too close to this patient or maybe the patient reminded them too much of mom or dad or grandma or grandpa. There have been providers who just needed a break. ‘I need to just take a step back’, you know? I remember we had a span where we probably had three or four within a three- or four-day period, on the same unit, which is a lot, and I think that team needed a break, a pause to reconnect with other things (Ethicist 2).*

Deciding whether to participate in MAiD often required HCPs to engage in introspective moral analysis. The many nuances accompanying a patient’s condition demanded deep emotional reflection and discernment from HCPs on a case-by-case basis. This type of reflection and discernment was necessary to determine not only their level of support for and involvement with MAiD but also to identify the possible emotional risks associated with engaging in MAiD.

*Lots of moral and ethical distress. Should we be there? Should we not be there? One of the things I was asked to discern, before even joining the team, was would it be okay for people to know that I was doing this. It was a discernment that I had to do to make sure that the people in my life were aware of because we didn’t know what to anticipate. I had lots of honest conversations (Nurse 3).*

*Although I really do want patients to have access to this service, it’s a really hard, emotional, awful thing to go through. I still get anxious and nervous (Physician 3).*

*When I had been kind of questioning whether I wanted to be involved, I think I was really afraid that MAiD was going to be scary and that it was going to be traumatic; not just for the patient and family but also for me (Physician 5).*

Participants also shared with us that their decision to participate in MAiD processes was influenced by several other factors including patient suffering; personal and professional values and beliefs; relationships with colleagues, patients and family, and community; and changing MAiD policy and legislation. The interplay of these factors suggests that the nature of their decision-making was dynamic, fluid, and situational and as such, resided on a continuum. We provide a detailed discussion how each of these factors influence HCP experiences with participation in MAiD in the following sections.

### Patient suffering

Suffering is a deeply personal experience that is complex and nuanced. All the HCPs in this study indicated that they heard, and to some extent experienced with their patients, their suffering and that they accepted their patients’ suffering without judgement. “*I always say this to the patients. I tell my patients, ‘I’m not here to tell you you’re not suffering. You tell me you’re suffering. I’m not going to argue that. I’m not going to argue that your life is good’” (Physician 7).*

While participants accepted that their patients were suffering, the participants also attached meaning to that suffering. Consequently, many of the participants spoke of suffering in conjunction with what they perceived was a ‘bad death’: a prolonged dying in which the patient experienced extensive pain. As HCPs, these participants felt it was their responsibility to alleviate suffering as much as possible and MAiD was a vehicle to do that.

*We’ve been putting down animals, in humane ways, that we care about, for I don’t know how long. I grew up on a farm and I will never forget – I accidentally ran over a cat one time and I was devastated. My dad came out with the gun and he was like “this is sad and it’s scary and I know you’re young, but this animal is not allowed to suffer”. Animals, cows, and things that were down and were not going to recover [we were] taught that’s a way to be humane, and to try to not let things suffer. I think I carried that right through into med school where I saw a lot of bad deaths. I don’t want to watch these patients have these bad deaths and so I thought if there’s an option I can bring to them, it was something I thought I could help with. It’s what keeps me going because I find it meaningful (Physician 7).*

In the comment above, Physician 7 acknowledges that patients suffer and that healthcare professionals need to respond to that suffering with compassion. In the following comment, he also demonstrates that his decision to support and participate in MAiD was not solely based on the patient’s suffering; there were other factors that influenced his decision-making. From his perspective (and in accordance with the law) suffering was not enough to qualify for MAiD.

*There are a lot of people out there who are suffering. They don’t qualify for MAiD. I mean, I’ve had several patients where I just cannot qualify them as a serious incurable disease. So, it’s hard because these patients will keep telling you, “I hate my life. I’m tired of living. I’m suffering.” I know you are. I understand that but without all this other stuff, it doesn’t qualify you for MAID. It’s really a problem for me. When I joined MAiD, I wanted to help patients die with dignity. I have no intention of being a mode of suicide. I’m not the gun; I’m not the gas; and I’m not the pills. I’m not interchangeable with those things (Physician 7).*

So, while patient suffering is an important factor in determining HCPs’ decision to participate in MAiD processes, tension between what quality of life and a good death mean, created nuances that influenced HCPs’ decision-making processes.

*Most practitioners – so someone who might not want to participate in MAiD still believes very much in the dignity of human life and a good death, they also don’t want someone to have a bad death. And then those who are, quote, “pro-MAiD” who also believe in the dignity of life, so I mean it’s not so much it’s one or the other, it’s where does the balance lie and what we emphasise over something else. So very nuanced (Ethicist 2).*

In the end, deciding to participate in MAiD was a relatively straightforward process for some of the rural HCPs in this study since it clearly relieved patient suffering. Though other HCPs shared this value and goal, they demonstrated more uncertain and evolving decision-making about their participation in MAiD. In sum, despite other factors like religious/spiritual perspectives, witnessing a patient’s irremediable and grievous suffering can also influence where a HCP lands on the continuum and their degree of willingness to participate in MAiD processes.

### Personal and professional values and beliefs

Unsurprisingly, personal values and beliefs (e.g., regarding patient autonomy, dignity, and fairness) informed many HCPs’ participation in MAiD. Values and beliefs are social in nature and are thus situational and time dependent. These values may also conflict with one another (e.g., respecting a person’s autonomy and what one believes is right or wrong). As such, the influence that personal values and beliefs have on one’s participation in MAiD can be fluid and context specific, as described by a nurse participant. For this participant, personal values were less important within the context of their professional relationship with patients and families.

*It’s my personal belief that it’s not my place to agree or disagree, like or dislike, influence – tell you yes or no, that you should or shouldn’t do this. It’s my place to respect your decision, your freedom of choice. So that’s why I don’t have an issue with my [Conservative and religious] upbringing and being part of the MAiD program. This isn’t about me and my beliefs, this is about allowing a person to choose their destiny, their lifestyle. I don’t have to like it or agree with it. But I dang well need to respect your personal decisions (Nurse 1).*

A professional value that nurses spoke about was the concept of ‘duty of care’. Although professional association guidelines and healthcare organizations in Alberta have been clear that participation in the assessment and provision of MAiD services is voluntary for NPs and physicians, we also heard that the responsibilities for provision of pre/post-care for RNs without NP designation are less clear, especially when leadership actively discourages any involvement. Consequently, some participants felt strongly that the duty of care pre/post provision still applied even if colleagues identified as conscientious objectors.

*It was always very clear that whether their beliefs were aligned with this process, or if they were a conscientious objector, we have a duty to provide care before and after (Nurse 3).*

*I run into issues of objection regularly. I have had some people in leadership positions who have been actively discouraging or even forbidding their staff to be involved in MAiD. It is often difficult to go around those barriers. Getting them out of the way is next to impossible in the moment and it causes a lot of stress during routine, task-oriented patient care. Staff may say, “it’s my right to not be involved in any way whatsoever” but where do you draw the line in terms the effect on patient care and on those people who are willing to help when the workload can’t be shared equally? (Nurse 6).*

In sum, personal and professional values informed decisions to be involved or not with the MAiD program. In some cases, professional values were articulated in formal guidelines or codes of conduct. In others, they emerged in the relational context of the work. Personal values were also considered important for guiding decision-making, with an emphasis on both personal implications and the implications for one’s patients.

### Relationships with colleagues, patients and family, and community

Consistent with the extant literature pertaining to the characteristics of rural practice, HCPs in this study spoke of close professional and, at times, personal relationships they enjoyed with their colleagues. Set against the backdrop of being ‘it’ [42] where trust and respect among rural colleagues is central to the provision of quality care, it is perhaps not surprising that relationships with colleagues was a significant factor that influenced the participants decisions regarding participation/nonparticipation in MAiD. One participant spoke about how a colleague’s comments and community disapproval affected their experience of MAiD participation:

*My colleague called me the day of and told me they couldn’t believe that I was going to go out to the patient’s house and murder them. I thought, you know, ‘great, now this has happened, and I have to go and provide for this woman and her family who I know, and I have this voice of this physician in the back of my head’. It was hard. I ended up having a good frank phone call with the physician a few days later. It’s hard when we live in a community where MAiD isn’t talked about very much, and where patients are afraid of asking, and where physicians who were quite closely involved with patients often will be very negative about it, rather than just remaining neutral. I would say the hardest part has been dealing with fear from people, and sort of the backlash of our somewhat conservative community (Physician 5).*

To that end, many participants spoke of the possible personal and professional repercussions they might experience because of their support for the program.

“*A few doctors are firmly based in their religion, and they feel it is really wrong to participate in MAiD. I didn’t ever want them to know that I was providing that service because it would have changed how they looked at me or felt about me, and maybe potentially how we were able to interact at work. This is Southern Alberta; it’s a small pond and we all kind of know each other: your choices can affect your professional opportunities down here if people don’t agree so it is something certainly that has a big effect (Nurse 2).*

*I was worried, people told me like, ‘your kid won’t get invited to birthday parties,’ stuff like that. So it was nerve-wracking (Physician 1).*

In fact, some participants felt that repercussions could be severe enough to impact their livelihood. Therefore, declining or reconsidering participation in the program was not because of conflict in personal or professional values, or based on religious or ideological convictions, but rather because they had a very practical concern of being able to continue to practice in that setting.

*You also have staff who are wanting to be involved in supporting MAiD patients, who then feel that they are not able to do that because they know that their leadership does not support it. This is a major difficulty in that you have people who would be very well suited to this work, but who feel that it is not good for their career or their job to become involved (Nurse 6).*

Although involvement in MAiD had the potential to create tension between their colleagues and themselves, some participants explained that with time and education, trust was built when safeguards were implemented that respected everyone’s beliefs.

*They [referring to palliative care physician colleagues] were afraid that I would make it too easy for patients to get MAiD. They said, ‘we’re afraid you’re putting people on the escalator to get it, to end it.’ I was like, ‘you want me to put these really frail people and make them go up the stairs?’ So, I had to make an agreement that we would ask all patients to self-refer to me so that it was clear it wasn’t coming from me. As time evolved, that’s softened. So now I still often have people self-refer, but the team will sometimes make referrals or if they’re not comfortable, they’ll tell me and they’ll ask me to refer (Physician 1).*

Like the close relationships they shared with colleagues, many participants explained that they also had personal and/or professional relationships with patients and their families in their rural communities. These relationships were often perceived as being a positive factor that helped participants feel more comfortable with providing MAiD. Comfort and conviction were amplified when it was felt that patients not only wanted MAiD, but also wanted it from someone who was known and trusted:

*I tend to only do usually my own patients, or I will take referrals from my colleagues. I think as family doctors, we get to be part of that true cradle to grave medicine, quite literally in this circumstance. It’s funny because there are still family doctors that think that like, ‘oh, well maybe my patient wants a specialist to do this or whatnot.’ But when you ask patients, they have that trusting long-term relationship with their family doctor. For the majority of them if their family doctor thinks they can do something, they would rather have their family doctor then a specialist (Physician 3).*

*I have provided for at least a small handful of people I know. I have assessed people that I know. I am always cautious to say that there’s other assessors, but it’s interesting. The patients want it. It does not throw the patients off (Physician 7).*

In fact, some participants indicated that the absence of a personal connection decreased their comfort level and could move them along the continuum to a point where they felt they would or could not provide the service.

*It was hard being the nurse that went in having no relationship with them because I’d have to show up at the house and do things that weren’t warm and fuzzy, a little bit sterile. You have to start an intravenous, you have to get paperwork done, you have to put the MAiD tag on and things like that. I felt like it was hard to go in and do these things with a family and with patients and not have any relationship. It was kind of uncomfortable I think for them to have this stranger come into their home and be a part of this journey that you’d just met at the door (Nurse 2).*

*I think you must have a personal rapport. I really do try, otherwise I wouldn’t do it. If it’s a push-button, no thanks. I’d sort of like to know a little bit about the person and be able to pet their dog or exchange some personal information or share something with them. I think it’s important. Otherwise, you’re just an executioner instead of a helper (Physician 4).*

*I had done MAiD provisions for people that I don’t know, and I much prefer doing MAiD provisions for people who I have seen for something other than their assessment. I think it’s very different when I know the patient already and I can walk in and I’ve already met some of the family, at least someone and they feel comfortable inviting me into their house and I feel comfortable in their space. I want people to feel safe and comfortable when I’m there in their presence, so I don’t seem like this scary person coming to cause their family member to die (Physician 5).*

Our findings indicate that decision-making about MAiD was highly relational for the HCPs in our study. Their relationships with their colleagues, their patients, and members of the broader rural community influenced their decision to participate in MAiD. Moreover, given the varied nuances that existed within their relationships, their decisions necessarily had to be fluid and situationally informed, further reflecting the continuum of participation in MAiD.

### Changing MAiD policy and legislation

For some participants, the possibility of new MAiD legislation that expands eligibility beyond those whose death is reasonably foreseeable, was welcomed and affirmed their decision to participate in the program.

*I would like to see legislation expanded to include people’s personal directives and wills. I think too often we lose sight of not every person, not every situation, not every single guideline is in stone. We need to be flexible, adaptable, look at everything because every person is different, every situation is different. I just want to see some more flexibility, more access to it, less barriers to it (Nurse 1).*

*I have always thought that we should be providing MAiD to patients with severe mental illness; the introduction of [Bill] C-7 is sort of the step in that right direction. I think it takes so much effort to go through the process of being assessed for MAiD, especially in the context of not having a reasonably foreseeable death. If someone is just flippantly “Yeah, maybe this is better; I don’t have enough money to live so I might as well die”, no one’s going to approve someone on that basis. I’ve met people who are so horrifically depressed and have tried and tried and they have no quality of life, I would much prefer to provide them with MAiD in a comfortable and safe setting with loved ones around them than to see them die by suicide. I think they [referring to lawmakers] forget the human nature behind this; that there are those of us who do assessments who actually sit down with the patient and actually genuinely hear their story and try to figure out if MAiD is appropriate for them. It’s not a bureaucrat in the office checking boxes saying, “Yeah, you want to die, and you filled out the paperwork, so we’re done”. I think because there are so many processes in place, if someone genuinely wants to apply for MAiD who is not reasonably foreseeable to die, I think it can be done. There will be people who have no quality of life and that’s why I’m a firm believer that we should be considering MAiD for people with severe psychiatric illness. So, I don’t really have issues with the legislation, and I think it’s moving in the right direction (Physician 5).*

For other participants though, the expanded eligibility criteria in Bill C-7 created uncertainty for them since the change seemed to undermine their core personal and professional values and corresponding actions. Consequently, having adequate safeguards as the legislation evolves seemed to be an important factor for HCPs to determine where on the continuum, they would be morally comfortable.

*With the new legislation, wow! It’s just – it really made me think about whether I’m going to continue to do this because it can be very time consuming, and very opinion based. It’s [referring to the introduction of Bill C-7] brought up things like do you know what a foreseeable death is because we’re all going to die. Is that like a specific timeframe? I think the court so far has said we’ll trust the physician’s judgement. But man, like this could – you can get one bad apple and it will really destroy this pathway which I think for people who are suffering I believe that this is a lovely option. But we allow too many groups to access this I can see it just going completely…I mean there are people that would fall under C-7 that I would support in terms of MAiD, but there are some physicians who wouldn’t. Then there are some people that other physicians would qualify but I wouldn’t. There was actually one lady that I wouldn’t approve. She eventually got approved but I said I would absolutely not do the provision. I just felt like I would be going against my own moral decision (Physician 2).*

*I’m nervous [referring to the introduction of Bill C-7]. I’m scared. I don’t know how I’m going to integrate this, because I mean I know I can’t be forced to do anything, but I’m a little concerned what it’s going to look like. Sure, this person meets the criteria, but I’m not doing it. Like, I’m just not. I don’t know how that’s ever going to balance out from a human rights perspective, but if they meet the criteria and I’m going ‘well, I’m not comfortable with this one, but that one I am,’ I really don’t know. I am very, very anxious about where things are going. Reasonably foreseeable death never hung me up a whole lot, but that was because, we’re all dying. But I still was mostly dealing with patients who had, if not – like, not three months, not six months, could’ve even been longer than that, but this thing is going to kill them. I’m really struggling with the not reasonably foreseeable deaths (Physician 7).*

*I think that for some people [expanding the eligibility criteria] won’t meet the need soon enough and I think for others, they will be uncomfortable with the permissiveness of it. As we evolve, we will have to be cognisant of the safeguards. (Nurse 3).*

*A patient needs to ask for it [MAiD], they still must have a life-limiting diagnosis, and if there’s any concern that they are suffering from significant depression, and I feel that that would be pushing it, I would involve other people. I guess that’s my comfort with mental illness. If mental illness is not your only diagnosis, then yeah, I think it’s an option for you and you need to understand what you’re asking for. [But having only a mental illness] will be tough because how do we figure out if they’re just suicidal and they want an out or they are undertaking treatment and the meds don’t fix it? It’ll be tough in some situations (Nurse 4).*

*Some physicians are not doing assessments or provisions for people who do not have that clear disease that’s going to end their life. But I have decided for myself, at this point, that I will not do a provision on anybody unless I’m absolutely comfortable (Physician 2).*

Overall, our participants did not view the provision of MAiD services as binary, as something that one either opposes or supports. In their discussions with us, it was clear that multiple factors influenced their decisions. Furthermore, existing nuances, the particulars of the law, and the evolving eligibility criteria further influenced their decisions.

### Discussion & implications

Decisions about participation in MAiD involve deep reflection and discernment. Like Haining and Keogh’s [35] conclusions, our participants’ degree of participation was contingent on their interpretation of the moral acceptability of the end-of-life activities as well as contextual factors like social interactions with colleagues, patients and family, community members, and the rural context [30, 31]. Participants described the dynamic nature of their decision-making that seem to reflect a threshold of acceptable participation: in certain contexts and under certain conditions where they were comfortable providing the full range of MAiD services and at other times, only certain services or no services at all.

Broadly speaking, reasons for when they choose to participate, limit the degree of their participation, or engage in nonparticipation in MAiD processes were rooted in conscience and non-conscience-based reasons. Personal values and ethics and the need for moral coherence created conscience-based reasons and, legal and professional risk, patient factors, personal competence, use of other end-of-life care options, and emotions created non-conscience-based reasons [43]. Moreover, as Brown et al. suggest in their work [3, 32], the participants in this study indicated that as they engaged in and integrated new personal and professional knowledge and were exposed to new experiences, their perspectives and actions changed. For example, scenarios where ‘a reasonably foreseeable death’ was unclear seemed to present moral contexts that created uncertainty for some participants. It may be possible that evolving legislation and the intertwining of professional responsibility and moral ideals (such as do no harm) found in ethical codes and professional duty beliefs resulted in blurring obligations for them [3, 32]. We suggest then, as legislation continues to change over time, HCPs should complete values-based assessments to clarify or deepen their understanding of their own ethical perspectives regarding MAiD [44] and to engage in personal and professional moral discovery through reflective dialogue with colleagues, workplace leaders, professional associations, and their community [30, 31]. The values-based assessment tool suggested by AHS [44] involves four steps: phase one is a review of phases that may lead to MAiD and HCPs roles in each phase; phase two is a values clarification exercise; phase three asks participants to consider out of six possible perspectives that outline degrees of participation in MAiD, given their values, which perspective they align most with and; phase four presents implications for practice associated with each perspective outlined in phase three.

Participants also spoke of having similar foundational beliefs as objectors regarding living with dignity and dying with dignity. However, some participants described instances of ‘them against us’ scenarios where they supported and participated in MAiD processes but that colleagues, leaders within their facility, and community members expressed opposition to MAiD. At times, their personal and professional relationships with colleagues as well as perceived personal and professional consequences had the potential to inform their decisions to support and participate in MAiD processes. It is perhaps necessary therefore, that HCPs contemplating participating in MAiD determine the perceived level of risk they are comfortable taking on.

To that end, we believe that language in policies, laws, guidelines, reports, and popular media may entrench binary positioning and make MAiD services polarizing. It may also create stereotypes in which providers are described as ‘heroes’ or ‘killers’ [31] that inadvertently create ‘secret-societies’ [45] in which participation in MAiD programs is silenced. We suggest that to create a more inclusive, responsive, and culturally sensitive environment, open discourse based on cultural awareness where there is recognition of diverse opinions on ethical issues [29] is necessary. This approach would need to be rooted in a critical analysis of the language found in professional and public MAiD discourse. We also suggest that all players (that is, policy and lawmakers, leaders in healthcare and healthcare practitioners, community and religious leaders, and researchers) join in discourses that include a discussion of nuanced factors that reflect the full spectrum between objection to and participation in the provision of MAiD.

Participants also perceived safeguards such as checklists, documentation, and guidelines as being helpful for ensuring they could support MAiD in good conscience and provide lawful services. That said, there was also uncertainty regarding how effective those safeguards would be as the legislation evolves and incorporates broader eligibility criteria. To be contextually relevant and sensitive to the evolving nature of the legislation, we agree with Lamb et al’s [27] recommendation that policies need to avoid challenging HCPs conscience. Safeguards, therefore, must support HCPs duty to care as well as their rights to health and conscience regardless of where HCPs find themselves on the continuum. In other words, documentation forms and guidelines, and responsibilities for communicating one’s position on the continuum described earlier should avoid identity-based framing (i.e., being a supporter or objector). Instead, framing of such documents and conversations should reflect ongoing decision-making processes and recognize that actions are contextual, fluid, and situational. We encourage HCPs to engage directly in discussion with workplace decision-makers and legislative policy makers, both as individuals and through their professional associations. This could help to ensure that those in positions of decision-making authority are attuned to the ethical nuances faced by those with direct lived experience. We also encourage leaders to be open to HCPs’ experiences and what MAiD means to them by establishing processes that allow HCPs to discuss where they may fall on the continuum at a given point in time. This support could also include providing regular debriefing opportunities, offering educational sessions regarding changes in legislation and related responsibilities, and engaging HCPs in discussions about staffing logistics and MAiD accessibility.

### Limitations

While many studies examine physicians or nurses’ experiences independently, we chose to interview general rural practice physicians, NPs, RNs, and ethicists. A limitation is that some groups who may be involved with MAiD (such as pharmacists or social workers) were not interviewed. Future research would benefit from the inclusion of these perspectives, particularly since nonparticipating pharmacists can have considerable implications for the logistics of rural MAiD provision [45].

Because our recruitment criteria included “individuals who have experience with MAiD provision”, the participants in this study supported MAiD and participated to some extent in its’ related processes (i.e., nursing care, assessments, and/or provisions) as per their professional scope of practice. While it may be tempting to describe these participants as simply “MAiD supporters”, their stories are heterogeneous and their perspectives are nuanced. We acknowledge, however, that interviewing HCPs who identify as conscientious objectors would deepen our understanding of that end of the continuum.

We collected our data during the Omicron wave of the COVID-19 pandemic which necessitated interviews be conducted via Zoom and telephone. Although telephone and virtual interviews have limitations since there may be less time to establish rapport, and telephone interviews preclude picking up on non-verbal cues, we did not have any issues with internet connectivity or in establishing rapport. Indeed, given the ubiquitous use of virtual communication platforms like Zoom during this time, participants seemed quite comfortable and familiar with the format, which made it easy to connect and engage in rich conversations.

## Conclusion

The findings of this study provide a glimpse into rural Albertan HCPs’ experiences within the context of MAiD. The participants’ experiences suggest that the decision to participate in or object to MAiD is not a binary one but rather is nuanced thereby reflects a continuum. Moreover, the decision and threshold to participate in, provide limited support, or engage in nonparticipation is influenced by internal (conscience-based) and external (non-conscience-based) factors. Lastly, evolving legislation might have significant practice implications for HCPs and ultimately, Canadians. Open discourse underpinned by inclusive language and responsive action to the changing landscape will ensure that HCPs, decision-makers, and patients and their families are respected, and needs are met.

### Electronic supplementary material

Below is the link to the electronic supplementary material.

Supplementary Material 1

## Data Availability

The datasets generated and/or analyzed during the current study are not publicly available due to participant confidentiality.
