# Supplementary material for: Rural healthcare professionals’ participation in Medical Assistance in Dying (MAiD): beyond a binary decision
Source: BMC Palliat Care. 2024 Apr 25;23:107. doi: 10.1186/s12904-024-01440-4 (PMC11044390; doi:10.1186/s12904-024-01440-4)
Supplement: Supplementary file 1 — Supplementary Material 1 [file 12904_2024_1440_MOESM1_ESM.pdf]

Interview Guide for Healthcare Professionals

1. Where do you currently practice?
    - How long have you been working there?

2. You indicated when arranging this interview that you have experience with proving care for a MAID client. About how many MAID clients have you cared for?
    - Can you tell me a bit about their journey?
    - What was your involvement like?

3. There has been some literature published on the implications of MAID for healthcare professionals providing end of life care. What kinds of discussions or debates have come up in your life or your workplace?

4. What are some of the biggest challenges that you've encountered in providing care for a MAID client?

5. What aspects of MAID consultation and provision do you think are working well in the province?
    - What have been the positive aspects of your experience?

6. Some scholars have expressed concerns that MAID service provision may play out differently in rural areas, specifically related to things like physician/nurse/nurse practitioner shortage, dual roles, less privacy or anonymity, and geographic isolation. Based on your experience, do you think that any of these concerns are warranted? Why or why not?

7. Have you seen any differences in knowledge/attitudes about MAID between urban and rural clients? Rural health providers?

8. As the MAID legislation continues to evolve, are there any policy changes that you think we need to see at federal and/or provincial levels?
    - With the passage of Bill C-7 and the expanded eligibility criteria, there have been media articles about concerns that the criteria are too permissive and might allow people to receive MAiD for reasons associated with a lack of supports (palliative care, insufficient income, disability, etc.). Have you encountered this concern in your practice? Do you think that C-7 has sufficient safeguards to address this?

9. Is there anything that we haven't asked that you think we should know about providing care for MAID clients in rural Alberta?
